# Therapeutic Exercises During Hospitalization in Oncohematological Patients: A Randomized Clinical Trial

**DOI:** 10.3390/healthcare13192526

**Published:** 2025-10-06

**Authors:** Bruna Cunha de Souza, Cintia Freire Carniel, Juliana Zangirolami-Raimundo, Rodrigo Daminello Raimundo

**Affiliations:** 1 Laboratório de Delineamento de Estudos e Escrita Científica, Centro Universitário FMABC, Santo André 09060-650, SP, Brazil; bruna.cunha@aluno.fmabc.net (B.C.d.S.); cintia.carniel@fmabc.br (C.F.C.); 2 Departamento de Saúde da Coletividade, Centro Universitário FMABC, Santo André 09060-650, SP, Brazil; juliana.raimundo@fmabc.net

**Keywords:** hematologic malignancies, hospitalization, exercise therapy

## Abstract

**Background/Objectives:** Therapeutic exercises during hospitalization may provide important benefits for onco-hematological patients. However, the most effective protocols and outcomes for evaluation remain unclear. The objective of this study was to evaluate the effects of a structured exercise program during hospitalization. **Methods:** We conducted a randomized clinical trial with hospitalized onco-hematological patients. The control group performed conventional exercises, while the intervention group received a combined program of aerobic and resistance training. Outcomes included functional capacity, muscle strength, quality of life, and fatigue, assessed at admission and discharge. The sample size was calculated for a moderate effect size (Cohen’s d = 0.50; α = 0.05; power = 80%), yielding a target of 35 participants per group. Data were analyzed using repeated measures analysis of variance, followed by Bonferroni post hoc tests. The significance level was set at 5%. **Results:** The intervention group showed significant improvements in dyspnea (*p* = 0.017) and pain (*p* = 0.024) compared with the control group. In addition, reductions in insomnia (*p* = 0.021) and improvements in emotional functioning (*p* = 0.024) were observed. No significant between-group differences were found for fatigue, muscle strength, or functional capacity. **Conclusions:** A short-term program of aerobic and resistance exercises was safe and improved pain and dyspnea in hospitalized onco-hematological patients, with additional favorable effects on insomnia and emotional function. However, no significant effects were detected in fatigue, muscle strength, or functional capacity, likely due to the short hospitalization period and limited number of sessions. Future studies should consider longer interventions and post-discharge follow-up to clarify the sustainability of these benefits.

## 1. Introduction

There are over 100 different types of cancer, each with distinct characteristics, and they must be carefully evaluated so that appropriate treatment can be administered. Considering the high incidence of the disease, all healthcare professionals should have consistent knowledge to treat their patients appropriately. To offer cancer patients appropriate management for their treatment, it is necessary to include a wide range of technical and care areas, including physical therapists, seeking an integrated multidisciplinary approach [1].

These patients may experience disabilities due to disease progression or treatment, particularly those with hematological neoplasms. In this context, the role of the physical therapist is highlighted in promoting the functionality, independence, and quality of life of cancer patients using various techniques, including the application of therapeutic exercises [2].

In recent decades, there has been an increase in the number of studies correlating cancer with physical activity, with evidence showing that physical activity is not only important for reducing the risk of developing certain cancers but also helps survivors recover from treatment and increases survival rates [3].

Research conducted with patients with hematological malignancies has shown that an appropriate exercise protocol, as well as regular exercise during hospitalization, can contribute to muscle strength gains, in addition to improving various functional aspects, emphasizing the need for protocols adapted to the patient’s hematological profile and clinical condition [4].

Early introduction of exercise in hematological malignancies helps combat sedentary lifestyles and maintain activity at any disease stage, provided the patient’s condition is respected [5,6].

The hospitalization of patients with hematological magnificence represents a critical period, marked by clinical complications resulting from both the disease and the adverse effects of treatment, such as fatigue, pain, loss of muscle mass, and decline in physical functional capacity [5]. Therefore, therapeutic exercises emerge as a promising intervention strategy, since they can reduce the deleterious effects of immobility and contribute to functionality even in conditions of prolonged hospitalization [7,8]. Recent studies show that structured exercise programs, applied under supervision during hospitalization, are feasible and safe, with the potential to improve quality of life-related symptoms, reduce fatigue, and maintain the level of physical activity of these patients [9,10].

An exercise program should be implemented in this population, either under the supervision of a professional or through guidance, if professionals know how to identify the specific characteristics of these diseases. It is important that an initial assessment be made to identify whether the patient is in a clinical condition to perform the activity, after that, specific exercises divided into phases, such as warm-up, training, and recovery, should be applied [5].

All forms of physical activity are important, including low-intensity activities [11]. Physical activity can be included at the beginning and end of treatment, but also during chemotherapy, both in the induction phase and in patients receiving high doses of chemotherapy drugs [7].

Therapeutic exercises can be prescribed to improve treatment, prevent harmful effects from treatment or disease, and improve patient outcomes in general [9]. To this end, it is necessary to conduct research that not only demonstrates the effectiveness of physical exercise, but also validates the type, amount, and duration necessary to improve patients’ quality of life [12]. Therefore, this study aims to evaluate a therapeutic exercise protocol in oncohematological patients during hospitalization.

## 2. Materials and Methods

This research was conducted following the CONSORT (Consolidated Standards of Reporting Trials) method [13] (Supplementary Materials).

### 2.1. Study Design

This randomized clinical trial was conducted with hospitalized onco-hematological patients who participated in an exercise protocol. Data collection was carried out prospectively, beginning in August 2022 and concluding in May 2025.

### 2.2. Participants

Adult patients aged 18 years or older were selected, who were hospitalized, had been diagnosed with malignant hematological disease, were in a clinical condition to perform the therapeutic exercise protocol, and had signed the Informed Consent Form (ICF). Data collection was carried out in the adult oncology ward of the Mário Covas Hospital in Sao Paulo/Brazil. The study protocol was submitted and approved by the Research Ethics Committee (#5.459.723). This study was registered in the Brazilian Registry of Clinical Trials (ReBEC). The clinical trial registry number is RBR-373nf9j (date of registration: 3 August 2023).

Patients with clinical conditions such as neurodegenerative diseases, heart failure, metastases in the central nervous system, and any other condition that prevents adequate communication and understanding, as well as patients who refuse to participate, were considered ineligible to participate in the study.

### 2.3. Interventions

#### 2.3.1. Interventions Methods

Once all assessments were completed, participants were allocated to two distinct groups: control group and intervention group. Treatment began according to group allocation, with daily supervised sessions from admission to discharge. In both groups, physical therapy lasted approximately 25 min.

The protocol consisted of 8 to 10 repetitions of exercises for the upper and lower limbs, seeking to work the functional muscle groups, in addition to aerobic activity. The intervention group performed the following sequence of exercises: lower limb ergometer cycle (5 min); shoulder flexion with dumbbells (2 × 8−10); elbow flexion with dumbbells (2 × 8−10); knee extension with shin guards (2 × 8−10); and they will be encouraged to walk around. The control group performed: maximum inspiratory support (3 × 10); inspiration in times associated with upper limb elevation (3 × 10); diaphragmatic proprioception (3 × 10); and were encouraged to walk around.

In addition, patients who, at the time of consultation, presented signs and symptoms that made it impossible to perform the exercises and/or assessment, such as fever, vomiting, and diarrhea, or who reported not feeling well, did not undergo the protocol. The results of routine blood counts were also checked for participants; those with platelet counts equal to or below 10,000/μL and/or hemoglobin equal to or below 7 g/dL did not receive daily care.

The protocol was completed when participants were discharged from the hospital and underwent the same initial tests again to assess functional capacity, muscle strength, quality of life, and fatigue.

#### 2.3.2. Measurement Items

After recruitment at the oncology ward of the Mário Covas Hospital, the study participants signed the ICF and began testing to assess functional capacity, muscle strength, quality of life, and fatigue. Functional capacity was assessed using the 6 min walk test (6MWT), which consists of walking at a moderate pace, without running, for 6 min, counting the distance covered in meters. Isometric muscle strength was assessed using a manual muscle dynamometer (Instrutherm, São Paulo, Brazil). For verification purposes, finger flexion was standardized with the right upper limb flexed at 90 degrees while seated. Three checks were performed, and the best performance measurement was selected.

Quality of life was assessed using the EORTC QLQ-C30 (version 3.0), developed by the European Organization for Research and Treatment of Cancer. The instrument comprises five functional scales, three symptom scales, six single-item measures, and a global health/QoL scale, totaling 30 items. Scores are linearly transformed to a 0–100 scale according to the scoring manual. Higher scores on the functional and global health/QoL scales indicate better functioning and quality of life, whereas higher scores on the symptom scales/items reflect greater symptom burden [14].

Fatigue was assessed using the Multidimensional Fatigue Inventory (MFI), developed by Smets et al. [15], translated and validated into Portuguese by Baptista et al. [16], consisting of 20 items divided into five scales (general fatigue, physical fatigue, mental fatigue, reduced activity, and reduced motivation). The final result of each scale is calculated separately and can vary from 4 to 20, with a higher score indicating greater fatigue.

Before the start of the assessment and care, and immediately after completion, the following were checked: peripheral oxygen saturation (SpO2) and heart rate (HR) using a pulse oximeter (Nonin^®^, model 2500, Plymouth, MN, USA); and systolic (SBP) and diastolic (DBP) blood pressure measurements using a stethoscope (Littmann^®^, model classic II S.E., St. Paul, MN, USA) and a sphygmomanometer (BD^®^, Curitiba, PR, Brazil) on the upper limb, respiratory rate (RR), and pain perception was assessed using The Visual Analog Pain Scale (VAS). All measurements and values obtained during the vital signs check were recorded in the collection instrument, called the assessment form.

The intensity of the exercises was determined using clinical judgment (signs of respiratory distress or changes in vital signs) and the patient’s own report, and was adapted according to tolerance: reduction in intensity, duration, and number of repetitions, and use of the BORG scale, which should be maintained between 7 and 8. Maximum heart rate (HRmax) was constantly assessed using the formula: HRmax: 220 − age. Exercises were suspended if participants experienced nausea, dizziness, severe hypotension, heart rate above maximum during intervals, or pain with VAS two points above baseline during exercise.

### 2.4. Outcomes

The primary outcome of this study was the degree of fatigue. Secondary outcomes included quality of life, muscle strength, and functional capacity. All outcomes were assessed with validated instruments at admission and at hospital discharge.

We hypothesized that a combined exercise protocol, integrating aerobic and resistance training, would improve fatigue, muscle strength, functional capacity, and quality of life compared with conventional care during hospitalization. While not designed to test each exercise modality separately, we expected that the resistance component would contribute more directly to gains in muscle strength and functional capacity, whereas the aerobic component would have greater impact on fatigue and quality of life.

### 2.5. Sample Size

The sample size calculation was based on the primary outcome, change in fatigue scores assessed with the Multidimensional Fatigue Inventory. We adopted a moderate effect size (Cohen’s d = 0.50), with a significance level of 5% (α = 0.05) and statistical power of 80% (1 − β = 0.80). This parameter corresponds to the detection of a clinically meaningful difference between groups using repeated measures ANOVA, which was the planned primary statistical analysis. The choice of a moderate effect size is consistent with methodological recommendations and with previous rehabilitation and oncology trials, where such effects are considered clinically relevant [17,18]. Based on these parameters, the required sample size was estimated at 30 participants per group. To account for possible losses and dropouts during hospitalization, a 15% margin was added, establishing a recruitment target of 35 participants per arm.

### 2.6. Randomization, Allocation, and Blinding

The random sequence of interventions was carried out using a sealed opaque envelope. Participants were allocated into two groups: Group I (Intervention) and Group C (Control).

To ensure optimal implementation, the study provided separate treatment for each participant, and the evaluator was blinded to prevent communication between patients receiving different treatments. After data collection, participants were informed of which groups they had participated in.

It was not possible to blind the physical therapists who provided care because they had to follow protocol and the participant knew that there were two groups in the study, so only the evaluators were blinded.

This situation may indeed introduce performance bias, since patients and therapists were aware of group allocation. To minimize this risk, both groups were managed as similarly as possible in terms of clinical care, contact with staff during consultations was kept to a minimum, and outcome assessors remained blinded throughout the study, from baseline to final evaluation.

### 2.7. Statistical Analysis

The collected data were organized in Microsoft Excel spreadsheets and subsequently analyzed using the Statistical Package for the Social Sciences (SPSS), version 22.0 (Chicago, IL, USA). The normality of quantitative variables was assessed using the Shapiro–Wilk test. As all variables presented normal distribution, they were described by mean and standard deviation. Qualitative variables were summarized in absolute and relative frequencies. To characterize the sample, comparisons between groups were performed using Student’s *t*-test for quantitative variables and the Chi-square or Fisher’s exact tests for qualitative variables. All QLQ-C30 scales were transformed to a 0–100 scale and analyzed separately; no composite mean was computed across functional and symptom scales. To examine the effects of the intervention, a repeated measures analysis of variance (ANOVA) was performed, followed by Bonferroni post hoc tests for main and interaction effects. Effect size was also calculated using Cohen’s d for variables that showed statistically significant differences between groups and across time points. The significance level adopted in all analyses was set at 5% (*p* < 0.05).

## 3. Results

We hypothesize that an exercise protocol combining aerobic and resistance exercises will be able to improve muscle strength, functional capacity, quality of life, and fatigue, when compared to conventional care, during the hospitalization period.

**Figure 1:**
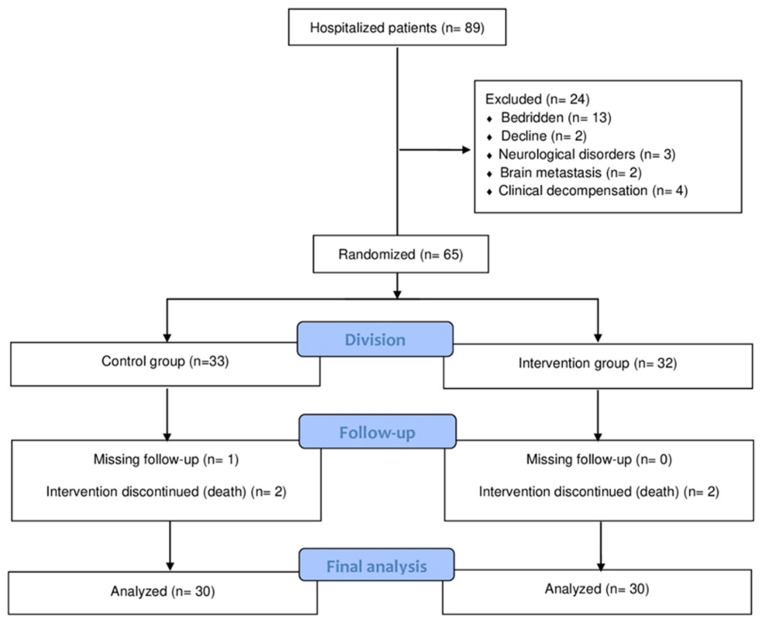
CONSORT Flowchart.

The total sample consisted of 60 participants who were hospitalized in the oncology ward of the Mário Covas Hospital and completed the clinical trial, as described in the flowchart below (Figure 1).

Among the 60 participants, the average age was 43 years, with chemotherapy being the main cause of hospitalization in both groups, with the number of visits ranging from 3 to 12, according to the data in Table 1.

Table 2 presents the participants’ diagnoses and the number of sessions performed during hospitalization. No statistically significant differences were observed between the groups for any of the variables presented in Table 2, confirming the comparability of baseline characteristics.

When analyzing physiological variables, muscle strength (dynamometry), and functional capacity (6MWT), no significant differences were found either within or between groups across the study period. Detailed values and post hoc comparisons are presented in Table 3.

The results obtained by the MFI showed that the intervention group had an increase in the “physical fatigue” domain score from 11.57 ± 3.18 to 12.53 ± 2.39 (*p* = 0.045). There was also an increase in the score in the “reduced motivation” domain in the intervention group, with a mean of 11.23 ± 3.61 and 12.13 ± 3.44 (*p* = 0.038). There was no difference between the groups in the two variables mentioned (*p* = 0.199 and *p* = 0.064, respectively).

There was a significant difference between the groups in the dyspnea and pain variables in the EORTC QLQ-C30 questionnaire.

In the dyspnea variable, there was a decrease in the score in the intervention group from 20 ± 31.07 to 7.78 ± 20.87, compared to the control group, with a score of 14.44 ± 32.88 and 16.67 ± 33.62, demonstrating a significant reduction between the groups (*p* = 0.017).

In terms of pain, the control group had scores of 30 ± 37.75 and 27.78 ± 40.19, while the intervention group had averages of 27.78 ± 34.56 and 8.33 ± 17.37, representing a significant reduction in pain between the groups (*p* = 0.024).

A reduction in the insomnia variable score was observed in the intervention group, from 50 ± 42.66 to 35.56 ± 40.05 (*p* = 0.021), with no difference between the groups (*p* = 0.371).

In the quality-of-life analysis (EORTC QLQ-C30), the intervention group showed a significant increase in the emotional functioning domain, with scores rising from 66.67 ± 29.36 to 75.83 ± 28.81 (*p* = 0.024). However, no significant difference was observed between groups in the comparative analysis (*p* = 0.239). Detailed results and post hoc comparisons are presented in Table 4.

The analysis of effect size indicated a moderate effect for dyspnea (Cohen’s d = 0.402) and a moderate-to-large effect for pain (Cohen’s d = 0.641).

## 4. Discussion

The present study evaluated the effects of a combined aerobic and resistance exercise protocol in hospitalized onco-hematological patients. Our initial hypothesis was that the intervention would improve muscle strength, fatigue, functional capacity, and quality of life compared with conventional care. The findings partially supported this hypothesis. Significant improvements were observed in pain and dyspnea, two domains of the EORTC QLQ-C30, in the intervention group. In addition, favorable changes were noted in insomnia and emotional functioning, as well as in the physical fatigue and reduced motivation subscales of the MFI-20, although these did not reach significance in the between-group comparisons. Conversely, no significant effects were found for overall fatigue, muscle strength, or functional capacity, suggesting that while exercise may alleviate specific symptoms during hospitalization, its impact on broader functional outcomes requires further investigation. These results should also be interpreted considering the methodological limitations and clinical heterogeneity of the sample.

Studies conducted with hematological patients using structured exercise protocols have demonstrated beneficial effects for participants. Patients diagnosed with lymphoma, leukemia, and multiple myeloma showed improvement in all subdomains of the EORTC QLQ-C30 questionnaire, a finding that corroborates the results of the present study, in which a reduction in pain and dyspnea scores measured by the same scale was observed [17]. It should be noted, however, that the quality of life of these patients is influenced by multiple factors, including biological and psychological aspects and those related to cancer treatment [19]. In this clinical trial, the authors did not identify any improvement in functional capacity, a result possibly associated with the difficulties encountered in applying functional tests to hospitalized patients, especially those kept in isolation [17]. In contrast, in the present study, no relevant limitations were observed for the application of the 6MWT in assessing functional capacity. However, despite the feasibility of the test, no improvements were observed in any of the groups analyzed.

Previous studies corroborate our findings, as seen by Streckmann et al. [20], when submitting patients diagnosed with lymphoma to an exercise protocol and assessing their quality of life, a significant difference was detected between the groups in the first 12 weeks; in addition, the control group showed no changes, while the intervention group showed improvement in quality of life, constipation, and a tendency toward improvement in pain.

The clinical trial conducted by Hacker et al. [21] using resistance training with patients undergoing chemotherapy and bone marrow transplantation showed that there was no difference in muscle strength after the protocol, but when analyzing fatigue (using the EORTC QLQ-C30 subscale), a decrease in fatigue was noted among participants in the group that underwent resistance training, compared to those who received only usual care. In our clinical trial, there was also no improvement in muscle strength, as assessed by the dynamometer, or in fatigue, as assessed by the MFI, but a significant reduction in dyspnea was observed in participants who performed resistance and aerobic exercises.

Another relevant aspect highlighted in the study by Hacker et al. [21] refers to the finding of worsening symptoms after transplantation, a factor that directly impacts the scores obtained in quality of life and fatigue assessment questionnaires. In addition, the authors observed an immediate reduction in muscle strength in these patients, as evidenced by specific tests, as well as a decline in physical activity levels.

In addition to adults, many children are also diagnosed with hematological malignancies, mainly ALL. This population was evaluated by Masoud et al. [22] and, although no differences were found between the groups evaluated, there was a significant reduction in all dimensions of fatigue and an increase in the level of physical activity in the intervention group, as well as an improvement in functional capacity and endurance.

It is important to note that hematological patients benefit from undergoing a structured exercise protocol, even during chemotherapy [22]. Accogli et al. [17] suggest that individual sessions, which are easier to implement, should be conducted initially, emphasizing that this type of hospital care for patients contributes to a more active life in the long term.

In our study, the intervention group showed an increase in the “reduced motivation” subscale of the MFI, which may initially appear to indicate a paradoxical or negative effect of the protocol. However, similar findings have been reported in hematologic populations undergoing rehabilitation. Fournié et al. [23] observed higher scores in the motivation domain after a combined physical exercise and biofeedback program, and Wiskemann et al. [24] also described increases in this subscale in both intervention and control groups following hospital discharge, although without statistical significance. These results suggest that the rise in “reduced motivation” may reflect the broader complexity of recovery in this population rather than a direct adverse effect of the intervention itself. Factors such as prolonged hospitalization, treatment burden, psychological distress, and uncertainty about reintegration into daily life are likely contributors. Given these contextual elements, we interpret the increase in “reduced motivation” not as a harmful effect of the exercise program, but as a signal of the multifactorial challenges faced by patients during post-treatment recovery. Nevertheless, this finding deserves further investigation in future studies to better understand its determinants and clinical implications.

The level of activity that these patients had prior to diagnosis and hospitalization should also be considered, since those with a higher level of previous activity tend to perform better and have better outcomes with the application of therapeutic protocols [25], although this difference was not identified in our study. Regarding fatigue, functional capacity, and muscle strength, no significant improvements were observed in our study. These results should be interpreted considering the short duration of hospitalization, which limits the number of intervention sessions available for each patient. In many cases, participants were exposed to only a few supervised sessions before discharge, which may not have been sufficient to produce measurable gains in these outcomes.

The literature also shows mixed findings in this respect. For instance, Masoud et al. reported positive effects on fatigue with a relatively short intervention [22], whereas Hacker et al. and Persoon et al. found that longer or more intensive programs were required to achieve improvements in fatigue and functional outcomes [21,26]. These discrepancies highlight the importance of factors such as intervention dose, program duration, and patients’ clinical status during hospitalization.

Taken together, our findings suggest that while in-hospital exercise interventions are feasible and safe, they may not always be sufficient to generate short-term improvements in fatigue, muscle strength, or functional capacity. Instead, they may serve as a foundation for continuity of rehabilitation after discharge, when longer intervention periods are feasible and potentially more effective. The effect size analysis reinforces the clinical relevance of the findings, with a moderate effect observed for dyspnea (d = 0.402) and a moderate-to-large effect for pain (d = 0.641). These magnitudes suggest that, although the intervention was delivered over a short hospitalization period, the benefits in symptom control may have practical implications for patient comfort and functional recovery.

Knowledge about the mechanisms by which physical activity acts in this population still needs to be clarified. Advances in this field will enable us to understand how exercise can contribute to improving clinical and functional outcomes, as well as guiding its most effective application, especially during hospitalization [9]. Furthermore, understanding the real effects of exercise on individuals with hematological diseases, including the impact on post-treatment symptoms, is a fundamental step toward broadening our understanding of the challenges faced in implementing rehabilitation programs [23]. It is also worth noting the relevance of such investigations given that this is a population that has been little explored in the literature, but which experiences significant functional losses, particularly during periods of hospitalization [27].

Despite the advances observed in recent years, including the demystification of contraindications traditionally attributed to the practice of exercise in this population—such as the risk of spontaneous bleeding during physical therapy appointments [28]—there remains a need for more robust clinical trials. Such studies should include larger and more homogeneous samples in order to consistently elucidate the real benefits of this therapeutic approach [29].

We must consider the length of hospitalization of patients, since in our study the maximum number of sessions was 12 in the control group and 10 in the intervention group. The duration of the protocols differs in the literature. There are studies that show gains in the analyzed variables after just a few weeks of follow-up. However, other studies present a protocol with a longer duration. This comparison can be seen in the study by Masoud et al. [22], with a protocol lasting 4 weeks, while Streckmann et al. carried out a protocol lasting 36 weeks [20].

The results of our study should be interpreted considering some limitations, including the heterogeneity of the sample in terms of age, diagnosis, and therapeutic protocols. Such heterogeneity may dilute the effects of exercise, since older patients, those with more aggressive neoplasms, or undergoing intensive treatments tend to show less pronounced gains compared to others. Our control group also performed conventional exercises. Although less specific, this type of activity may also have exerted beneficial effects, potentially reducing the differences observed between groups.

Furthermore, the relatively small number of participants in each group, with limited statistical power; the lack of stratification by disease stage or type of ongoing treatment; the variation in the number of consultations received during hospitalization; and the lack of follow-up after hospital discharge, which made it impossible to verify the maintenance of the observed effects in the long term, restricting its clinical applicability, can also be considered limitations.

It is important to recognize that the final study sample was limited by the number of eligible patients within the previously established recruitment period, which has now concluded. This limitation is characteristic of clinical trials involving hospitalized onco-hematological patients, in which factors such as isolation due to neutropenia, thrombocytopenia, nausea and clinical instability substantially reduce the inclusion rate. Several randomized studies in similar contexts also reported small sample sizes and/or recruitment difficulties [17,20,22,30,31]. Nevertheless, we consider that the sample obtained was adequate to respond to the main objective of evaluating the viability and preliminary effects of the intervention. 

The sample size calculation was based on an expected moderate effect on fatigue, but we recognize that the study may not have sufficient power to detect smaller, yet clinically relevant, effects. This limitation is especially pertinent in multifactorial outcomes, such as fatigue in hospitalized patients. We emphasize, however, that the estimate used is consistent with previous randomized trials in the area, which also adopted reduced samples due to the difficulty of recruitment in this clinical setting.

Despite the importance of post-discharge follow-up, which we acknowledge as a limitation and recommend for future studies, our findings highlight that patients were discharged with fewer complaints of dyspnea and pain. These improvements, even if measured only during hospitalization, may have facilitated their transition home and contributed to better short-term recovery.

## 5. Conclusions

Hospitalized onco-hematological patients may benefit from structured exercise interventions. In this study, a short-term protocol of combined aerobic and resistance exercises led to significant improvements in pain and dyspnea, with additional favorable effects on insomnia and emotional functioning. However, no significant benefits were observed for overall fatigue, muscle strength, or functional capacity, likely due to the short hospitalization period and limited number of sessions. These findings suggest that in-hospital exercise is feasible and may alleviate specific symptoms, while longer interventions and post-discharge follow-up are needed to clarify its broader effects on functional outcomes.

## Figures and Tables

**Table 1:** Baseline characteristics of intervention and control groups.

Variable	Control Group (*n* = 30)	Intervention Group (*n* = 30)	*p*
**DEMOGRAPHIC—N (%)**			
Age (years)	44.8 ± 17.14	41.7 ± 17.16	0.794
Weight (kg)	71.23 ± 15.03	73.20 ± 16.13	0.951
Height (cm)	170.43 ± 10.94	170.23 ± 9.54	0.906
**SEX—N (%)**			
Female	10 (33.3)	16 (53.3)	0.192
Male	20 (66.7)	14 (46.7)	
**REASON FOR HOSPITALIZATION—N (%)**			
Chemotherapy	27 (90)	27 (90)	1.000
Symptom management	1 (3.3)	1 (3.3)	
Biopsy and Chemotherapy	2 (6.7)	2 (6.7)	
**CHEMOTHERAPY—N (%)**			
Yes	28 (93.3)	28 (93.3)	0.195
No	2 (6.7)	2 (6.7)	
**PHYSICAL ACTIVITY—N (%)**			
Yes	18 (60)	20 (66.7)	0.789
No	12 (40)	10 (33.3)	
**NUTRITIONAL SUPPLEMENTATION—N (%)**			
Yes	17 (56.7)	11 (36.7)	0.195
No	13 (43.3)	19 (63.3)	
**NUTRITIONAL ASSESSMENT—N (%)**			
Yes	30 (100)	29 (96.7)	1.000
No	0	1 (3.3)	

**Table 2:** Participants’ diagnoses and number of physical therapy sessions during hospitalization.

Variable	Control Group (*n* = 30)	Intervention Group (*n* = 30)
**DIAGNOSIS—N (%)**		
ALL	5 (16.7)	10 (33.3)
NHL	9 (30)	8 (26.7)
HL	3 (10)	3 (10)
AML	10 (33.3)	4 (13.3)
CML	1 (3.3)	2 (6.7)
BAL	1 (3.3)	3 (10)
Multiple Myeloma	1 (3.3)	0
**NUMBER OF PHYSIOTHERAPY SESSIONS—N (%)**		
3	2 (6.7)	1 (3.3)
4	6 (20)	4 (13.3)
5	5 (16.7)	8 (26.7)
6	5 (16.7)	5 (6.7)
7	4 (13.3)	3 (10)
8	5 (16.7)	3 (10)
9	1 (3.3)	1 (3.3)
10	2 (6.7)	3 (10)
11	0	1 (3.3)
12	0	1 (3.3)

(ALL) Acute Lymphoid Leukemia; (NHL) Non-Hodgkin Lymphoma; (HL) Hodgkin Lymphoma; (AML) Acute Myeloid Leukemia; (CML) Chronic Myeloid Leukemia; (BAL) Biphenotypic Acute Leukemia.

**Table 3:** Physiological variables, muscle strength (dynamometry), and functional capacity (6 min walk test) in intervention and control groups.

Variables	Control Group			Intervention Group			(F)		
							P		
							(ƞ^2^p)		
	Before	After	*p*	Before	After	*p*	Moment	Group	Moment vs. Group
Distance Traveled (6MWT)	208.73 ± 109.00	220.50 ± 119.14	0.491	240.70 ± 146.81	255.43 ± 138.57	0.389	(1.220)	(1.154)	(0.015)
							0.274	0.287	0.902
							(0.021)	(0.020)	(0.000)
Dynamometry	18.42 ± 12.32	19.60 ± 15.24	0.480	19.1 ± 15.29	20.04 ± 15.99	0.573	(0.816)	(0.024)	(0.010)
							0.370	0.877	0.919
							(0.014)	(0.000)	(0.000)
HR in 2 min	87.13 ± 17.49	84.17 ± 16.23	0.366	95.30 ± 25.27	93.37 ± 17.68	0.555	(1.13)	(3.763)	(0.050)
							0.292	0.057	0.823
							(0.019)	(0.061)	(0.001)
SpO2 in 2 min	97.97 ± 1.35	97.53 ± 1.91	0.531	96.67 ± 4.60	97.20 ± 28.58	0.441	(0.01)	(1.936)	(0.990)
							0.918	0.169	0.324
							(0.000)	(0.032)	0.017
BORG in 2 min	0.77 ± 1.33	0.80 ± 1.37	0.921	1.47 ± 2.34	1.20 ± 1.65	0.426	(0.246)	(2.122)	(0.406)
							0.622	0.151	0.526
							(0.004)	(0.035)	(0.007)
HR in 6 min	99.33 ± 16.31	96.83 ± 15.86	0.442	106.50 ± 22.85	109.07 ± 18.93	0.430	(0.000)	(5.204)	(1.230)
							0.988	0.026	0.272
							(0.000)	(0.082)	(0.021)
SpO2 in 6 min	98.43 ± 1.38	98.00 ± 2.00	0.161	97.60 ± 2.25	97.70 ± 2.34	0.744	(0.596)	(1.412)	(1.527)
							0.443	0.240	0.222
							(0.010)	(0.024)	(0.026)
BORG in 6 min	3.63 ± 2.43	3.50 ± 2.65	0.746	3.90 ± 3.16	3.43 ± 2.64	0.260	(1.069)	(0.024)	(0.330)
							0.305	0.877	0.568
							(0.018)	(0.000)	(0.006)

(F) analysis of variance; (*p*) probability of statistical difference; (ƞ^2^p) Eta squared—effect size; (6MWT) Six-Minute Walk Test; (HR) Heart Rate; (SpO2) Peripheral Oxygen Saturation.

**Table 4:** Quality of life domains (EORTC QLQ-C30) in intervention and control groups.

Variables	Control Group			Intervention Group			(F)		
							P		
							(ƞ^2^p)		
	Before	After	*p*	Before	After	*p*	Moment	Group	Moment vs. Group
**Multidimensional Fatigue Inventory (MFI)**									
General Fatigue	9.93 ± 3.05	10.70 ± 2.78	0.188	10.47 ± 2.84	10.40 ± 2.80	0.908	(0.739)	(0.036)	(1.047)
							0.394	0.851	0.311
							(0.013)	(0.001)	(0.018)
Physical Fatigue	12.27 ± 3.43	12.37 ± 2.92	0.833	11.57 ± 3.18	12.53 ± 2.39	0.045	(2.553)	(0.145)	(1.686)
							0.115	0.705	0.199
							(0.042)	(0.002)	(0.028)
Reduced Activity	11.13 ± 3.13	10.70 ± 3.04	0.234	11.40 ± 2.44	10.73 ± 2.73	0.069	(4.657)	(0.047)	(0.210)
							0.035	0.829	0.649
							(0.074)	(0.001)	(0.004)
Reduced Motivation	12.37 ± 3.49	12.13 ± 3.66	0.585	11.23 ± 3.61	12.13 ± 3.44	0.038	(1.231)	(0.428)	(3.558)
							0.272	0.516	0.064
							(0.021)	(0.007)	(0.058)
Mental Fatigue	14.97 ± 3.12	15.30 ± 2.49	0.477	14.33 ± 2.60	15.00 ± 2.38	0.158	(2.306)	(0.596)	(0.256)
							0.134	0.443	0.615
							(0.038)	(0.010)	(0.004)
**EORTC QLQ-C30 (version 3.0)**									
Physical function	77.78 ± 19.76	78.00 ± 20.65	0.949	69.78 ± 27.11	75.56 ± 22.51	0.100	(1.511)	(0.962)	(1.295)
							0.224	0.331	0.260
							(0.025)	(0.016)	(0.022)
Functionality	51.11 ± 41.04	48.33 ± 44.06	0.677	52.78 ± 41.77	54.44 ± 42.65	0.802	(0.014)	(0.154)	(0.225)
							0.906	0.696	0.637
							(<0.001)	(0.003)	(0.004)
Dyspnea	14.44 ± 32.38	16.67 ± 33.62	0.593	20.00 ± 31.07	7.78 ± 20.87	0.005	(2.918)	(0.054)	(6.088)
							0.093	0.816	0.017
							(0.048)	(0.001)	(0.095)
Pain	30.00 ± 37.75	27.78 ± 40.19	0.675	27.78 ± 34.56	8.33 ± 17.37	<0.001	(8.468)	(1.901)	(5.350)
							0.005	0.173	0.024
							(0.127)	(0.032)	(0.084)
Fatigue	43.70 ± 30.31	45.93 ± 33.11	0.636	38.89 ± 34.62	30.74 ± 30.00	0.086	(0.807)	(1.734)	(2.471)
							0.373	0.193	0.121
							(0.014)	(0.029)	(0.041)
Insomnia	32.22 ± 41.51	25.56 ± 38.84	0.279	50.00 ± 42.66	35.56 ± 40.05	0.021	(5.999)	(2.089)	(0.814)
							0.017	0.154	0.371
							(0.094)	(0.035)	(0.014)
Appetite	32.22 ± 36.60	32.22 ± 40.57	1.00	37.78 ± 37.89	27.78 ± 37.22	0.077	(1.621)	(0.004)	(1.621)
							0.208	0.951	0.208
							(0.027)	(0.000)	(0.027)
Nausea	16.67 ± 27.68	14.44 ± 25.42	0.613	18.33 ± 28.48	19.44 ± 24.01	0.800	(0.032)	(0.299)	(0.292)
							0.858	0.587	0.591
							(0.001)	(0.005)	(0.005)
Constipation	31.11 ± 42.82	27.78 ± 41.14	0.656	26.67 ± 42.35	16.67 ± 32.46	0.185	(1.600)	(0.771)	(0.400)
							0.211	0.383	0.530
							(0.027)	(0.013)	(0.007)
Diarrhea	4.44 ± 19.04	7.78 ± 20.87	0.445	6.67 ± 20.34	10.00 ± 24.99	0.445	(1.184)	(0.233)	(0.000)
							0.281	0.631	1.000
							(0.020)	(0.004)	(0.000)
Cognitive Function	82.78 ± 20.75	83.89 ± 19.81	0.732	81.67 ± 29.15	85.56 ± 26.16	0.233	(1.200)	(0.002)	(0.370)
							0.278	0.962	0.545
							(0.020)	(0.000)	(0.006)
Emotional Function	70.00 ± 24.82	72.50 ± 27.17	0.530	66.67 ± 29.36	75.83 ± 28.81	0.024	(4.339)	(0.000)	(1.417)
							0.042	1.000	0.239
							(0.070)	(0.000)	(0.024)
Social Function	65.00 ± 33.72	62.22 ± 36.60	0.573	67.22 ± 33.47	67.22 ± 37.01	1.00	(0.161)	(0.184)	(0.161)
							0.690	0.669	0.690
							(0.003)	(0.003)	(0.003)
Financial Difficulties	27.78 ± 36.18	25.56 ± 35.75	0.674	41.11 ± 41.69	33.33 ± 41.06	0.144	(1.811)	(1.290)	(0.559)
							0.184	0.261	0.458
							(0.030)	(0.022)	(0.010)
General Health	63.06 ± 26.86	64.17 ± 22.76	0.771	65.56 ± 28.00	71.11 ± 26.69	0.149	(1.538)	(0.581)	(0.683)
							0.220	0.449	0.412
							(0.026)	(0.010)	(0.012)

(F) analysis of variance; (*p*) probability of statistical difference; (ƞ^2^p) Eta squared-effect size.

## Data Availability

The raw data supporting the conclusions of this article will be made available by the authors on request.

## Supplementary Materials

The following supporting information can be downloaded at: https://www.mdpi.com/article/10.3390/healthcare13192526/s1, CONSORT 2010 checklist.

## References

[1] Ministry of Health, José Alencar Gomes da Silva National Cancer Institute (INCA) (2020). ABCs of Cancer: Basic Approaches to Cancer Control. Rio de Janeiro.

[2] Almeida E.M.P., Andrade R.G., Cecatto R.B., Brito C.M.M., Camargo F.P., Pinto C.A., Yamaguti W.P.S., Imamura M., Battistella L.R. (2012). Exercise in cancer patients: Rehabilitation. Acta Fisiatr..

[3] Brazilian Society of Clinical Oncology, José Alencar Gomes National Cancer Institute (INCA) (2022). Physical Activity and Cancer: Recommendations for Prevention and Control. São Paulo.

[4] Longaray S.R.M., Oliveira D., Forgiarini S.G.I., da Silva V.G. (2021). Implementation of a Physiotherapy Protocol in Hematology-Oncology Patients. Braz. J. Cancerol..

[5] Bouanani N., Asly M. (2020). Adapted physical activity and hematological malignancies. Pan Afr. Med. J..

[6] Rosko A.E., Wall S., Baiocchi R., Benson D.M., Brammer J.E., Byrd J.C., Efebera Y.A., Maddocks K., Rogers K.A., Jones D. (2021). Aging Phenotypes and Restoring Functional Deficits in Older Adults with Hematologic Malignancy. J. Natl. Compr. Cancer Netw..

[7] Großek A., Elter T., Oberste M., Wolf F., Joisten N., Hartig P., Walzik D., Rosenberger F., Kiesl D., Wahl P. (2021). Feasibility and suitability of a graded exercise test in patients with aggressive hemato-oncological disease. Support. Care Cancer.

[8] Santos S.S., Moussalle L.D., Heinzmann-Filho J.P. (2020). Effects of physical exercise during hospitalization of children and adolescents with cancer: A systematic review. Rev. Paul. Pediatr..

[9] Sitlinger A., Brander D.M., Bartlett D.B. (2020). Impact of exercise on the immune system and outcomes in hematologic malignancies. Blood Adv..

[10] Schmidt M.E., Wiskemann J., Armbrust P., Schneeweiss A., Ulrich C.M., Steindorf K. (2015). Effects of resistance exercise on fatigue and quality of life in breast cancer patients undergoing adjuvant chemotherapy: A randomized controlled trial. Int. J. Cancer.

[11] Kowaluk A., Woźniewski M., Malicka I. (2019). Physical Activity and Quality of Life of Healthy Children and Patients with Hematological Cancers. Int. J. Environ. Res. Public Health.

[12] Schmid D., Behrens G., Arem H., Hart C., Herr W., Jochem C., Matthews C.E., Leitzmann M.F. (2018). Pre- and post-diagnosis physical activity, television viewing, and mortality among hematologic cancer survivors. PLoS ONE.

[13] Schulz K.F., Altman D.G., Moher D., CONSORT Group (2010). CONSORT 2010 statement: Updated guidelines for reporting parallel group randomised trials. BMJ.

[14] Aaronson N.K., Ahmedzai S., Bergman B., Bullinger M., Cull A., Duez N.J., Filiberti A., Flechtner H., Fleishman S.B., Haes J.C.D. (1993). The European Organization for Research and Treatment of Cancer QLQ-C30: A quality-of-life instrument for use in international clinical trials in oncology. J. Natl. Cancer Inst..

[15] Smets E.M., Garssen B., Bonke B., De Haes J.C. (1995). The Multidimensional Fatigue Inventory (MFI) psychometric qualities of an instrument to assess fatigue. J. Psychosom. Res..

[16] Baptista R.L., Biasoli I., Scheliga A., Soares A., Brabo E., Morais J.C., Werneck G.L., Spector N. (2012). Psychometric properties of the multidimensional fatigue inventory in Brazilian Hodgkin’s lymphoma survivors. J. Pain Symptom Manag..

[17] Accogli M.A., Denti M., Costi S., Fugazzaro S. (2022). Therapeutic education and physical activity are feasible and safe in hematologic cancer patients referred to chemotherapy: Results of a randomized controlled trial. Support. Care Cancer.

[18] Al-Majid S., Wilson L.D., Rakovski C., Coburn J.W. (2015). Effects of exercise on biobehavioral outcomes of fatigue during cancer treatment: Results of a feasibility study. Biol. Res. Nurs..

[19] Stan D.J., Tatu A.L., Lescai A.M., Popazu C., Vlad A.L., Dobrea G., Baltă A.A.Ș. (2025). Psychological Distress and Kidney Failure as Predictors of Chemoradiotherapy Toxicity and Quality of Life in Patients with Head and Neck Cancer. Healthcare.

[20] Streckmann F., Kneis S., Leifert J.A., Baumann F.T., Kleber M., Ihorst G., Herich L., Grüssinger V., Gollhofer A., Bertz H. (2014). Exercise program improves therapy-related side-effects and quality of life in lymphoma patients undergoing therapy. Ann. Oncol..

[21] Hacker E.D., Larson J., Kujath A., Peace D., Rondelli D., Gaston L. (2011). Strength training following hematopoietic stem cell transplantation. Cancer Nurs..

[22] Masoud A.E., Shaheen A.A.M., Algabbani M.F., AlEisa E., AlKofide A. (2023). Effectiveness of exergaming in reducing cancer-related fatigue among children with acute lymphoblastic leukemia: A randomized controlled trial. Ann. Med..

[23] Fournié C., Verkindt C., Dalleau G., Bouscaren N., Mohr C., Zunic P., Cabrera Q. (2022). Rehabilitation program combining physical exercise and heart rate variability biofeedback in hematologic patients: A feasibility study. Support. Care Cancer.

[24] Wiskemann J., Dreger P., Schwerdtfeger R., Bondong A., Huber G., Kleindienst N., Ulrich C.M., Bohus M. (2011). Effects of a partly self-administered exercise program before, during, and after allogeneic stem cell transplantation. Blood.

[25] Räder J., Ihorst G., Möller M.D., Pahl A., Greil C., Dreyling E., Arends J., Deibert P., Wäsch R., Engelhardt M. (2024). Physical activity and exercise motivation of multiple myeloma patients: A prospective cross-sectional study. Oncologist.

[26] Persoon S., ChinAPaw M.J.M., Buffart L.M., Liu R.D.K., Wijermans P., Koene H.R., Minnema M.C., Lugtenburg P.J., Marijt E.W.A., Brug J. (2017). Randomized controlled trial on the effects of a supervised high intensity exercise program in patients with a hematologic malignancy treated with autologous stem cell transplantation: Results from the EXIST study. PLoS ONE.

[27] Hacker E.D. (2019). Physical Activity and Exercise after Hematopoietic Cell Transplantation: Just Keep on Moving. Asia Pac. J. Oncol. Nurs..

[28] Morishita S., Nakano J., Fu J.B., Tsuji T. (2020). Physical exercise is safe and feasible in thrombocytopenic patients with hematologic malignancies: A narrative review. Hematology.

[29] Braam K.I., van der Torre P., Takken T., Veening M.A., van Dulmen-den Broeder E., Kaspers G.J. (2016). Physical exercise training interventions for children and young adults during and after treatment for childhood cancer. Cochrane Database Syst. Rev..

[30] Santa Mina D., Dolan L.B., Lipton J.H., Au D., Camacho Pérez E., Franzese A., Alibhai S.M.H., Jones J.M., Chang E. (2020). Exercise before, during, and after Hospitalization for Allogeneic Hematological Stem Cell Transplant: A Feasibility Randomized Controlled Trial. J. Clin. Med..

[31] Hacker E.D., Fink A.M., Peters T., Park C., Fantuzzi G., Rondelli D. (2017). Persistent Fatigue in Hematopoietic Stem Cell Transplantation Survivors. Cancer Nurs..

